# Hyperspectral reflectance integrates key traits for predicting leaf metabolism

**DOI:** 10.1111/nph.20345

**Published:** 2024-12-14

**Authors:** Troy S. Magney

**Affiliations:** ^1^ Department of Plant Sciences University of California Davis CA 95616 USA

**Keywords:** carbon cycle, hyperspectral reflectance, integrative traits, leaf dark respiration, photosynthesis, scaling, terrestrial biosphere models

## Abstract

This article is a Commentary on Wu *et al*. (2025), **246**: 481–497.

There has been widespread interest in developing trait‐based models to predict photosynthetic capacity from leaves to ecosystems (Walker *et al*., 2014; Xu & Trugman, 2021), but comparably less for nonphotorespiratory mitochondrial CO_2_ release (dark respiration, *R*_dark_). This is significant, given that about half of the CO_2_ released from plants is via *R*_dark_ – which occurs day and night – and supports ATP production, redox balance, nitrogen assimilation and carbon skeleton synthesis (Atkin *et al*., 2015). Terrestrial biosphere models use simplified empirical relationships between the maximum rate of carboxylation (*V*_cmax_) and *R*_dark_ – often derived from more easily measurable leaf traits such as leaf mass per area (LMA), leaf lifespan, nitrogen (N), and phosphorus (P), which have more extensive data availability (Reich *et al*., 1998; Tcherkez *et al*., 2024). Notably, these traits are measured across a unidimensional continuum, and there has yet to be solid evidence that the magnitude and direction of a leaf trait is highly predictive of a metabolic trait like *R*_dark_. Leaf metabolic parameters change dramatically with their environment and encompass an integrated suite of traits – some which increase, some which decrease, and some that remain unchanged. This begs the question – *is there an alternative approach*, *which integrates a large suite of the biochemical*, *structural and environmental traits*, *to predict R*_*dark*_ *on its own?* A paper published in this issue of *New Phytologist* (Wu *et al*., 2025; pp. 481–497) addresses this question by comparing the utility of traditional trait‐based approaches against hyperspectral reflectance data across three forest types.

*By incorporating bidirectional variations across the visible to shortwave spectrum, hyperspectral reflectance effectively captures dynamic shifts in a broad array of leaf structural and biochemical traits…*

Wu *et al*. (2025) show that while trait‐based models have provided valuable insights in some other studies, their predictive power of *R*_dark_ is underwhelming. The authors show that univariate trait*–R*_dark_ relationships are weak (*r* ^2^ ≤ 0.15), and even multivariate models explain only a fraction of the observed variability (*r* ^2^ = 0.30), leaving much of *R*_dark_ complexity unexplained. Beyond traditional leaf economic traits like LMA, N, and P, the authors investigate other elements such as magnesium (Mg), manganese (Mn), calcium (Ca), potassium (K), and sulfur (S), as they play crucial roles in respiratory metabolism but are rarely incorporated into predictive frameworks (Tcherkez *et al*., 2024). Despite the inclusion of more leaf traits for *R*_dark_ prediction, their poor performance highlights the need for alternative approaches that can more holistically capture the physiological complexity of *R*_dark_.

By incorporating bidirectional variations across the visible to shortwave spectrum, hyperspectral reflectance effectively captures dynamic shifts in a broad array of leaf structural and biochemical traits, offering a rapid, scalable solution for characterizing physiological variability (Fig. 1; Ustin *et al*., 2009). Using data from Wu *et al*. (2025), there are subtle differences between the mean, lowest 10^th^ and highest 90^th^ percentile of *R*_dark_ samples (Fig. 1a). To understand the magnitude and direction of spectral changes between low *R*_dark_ (10^th^ percentile) and high‐*R*_dark_ (90^th^ percentile) measurements, the percent difference from the mean spectra in the dataset is shown (Fig. 1b). In the visible spectrum, there is comparably less reflectance in the blue and red regions of the spectrum for high‐*R*_dark_ measurements, associated with chlorophyll absorption features. Additionally, a change in the opposite direction occurs in the green region – centered *c*. 531 nm – which has been shown to be sensitive to photoprotective carotenoid pigments, that is the xanthophyll cycle (Gamon *et al*., 1992). Beyond this, we observe differences in the near infrared (NIR), indicating leaves with higher *R*_dark_ are likely thicker, or have higher LMA, but might also have higher leaf water content – as is highlighted by changes in water absorption features in the shortwave infrared (SWIR). While specific nutrients do not have an explicit spectral signature, it is likely that their concentration covaries with these leaf biochemical and structural attributes (Wong, 2023). Taken together, increases and decreases across the spectrum seem to match what we would theoretically assume for leaves with greater photosynthetic capacity, and potentially higher *R*_dark_.

**Fig. 1:**
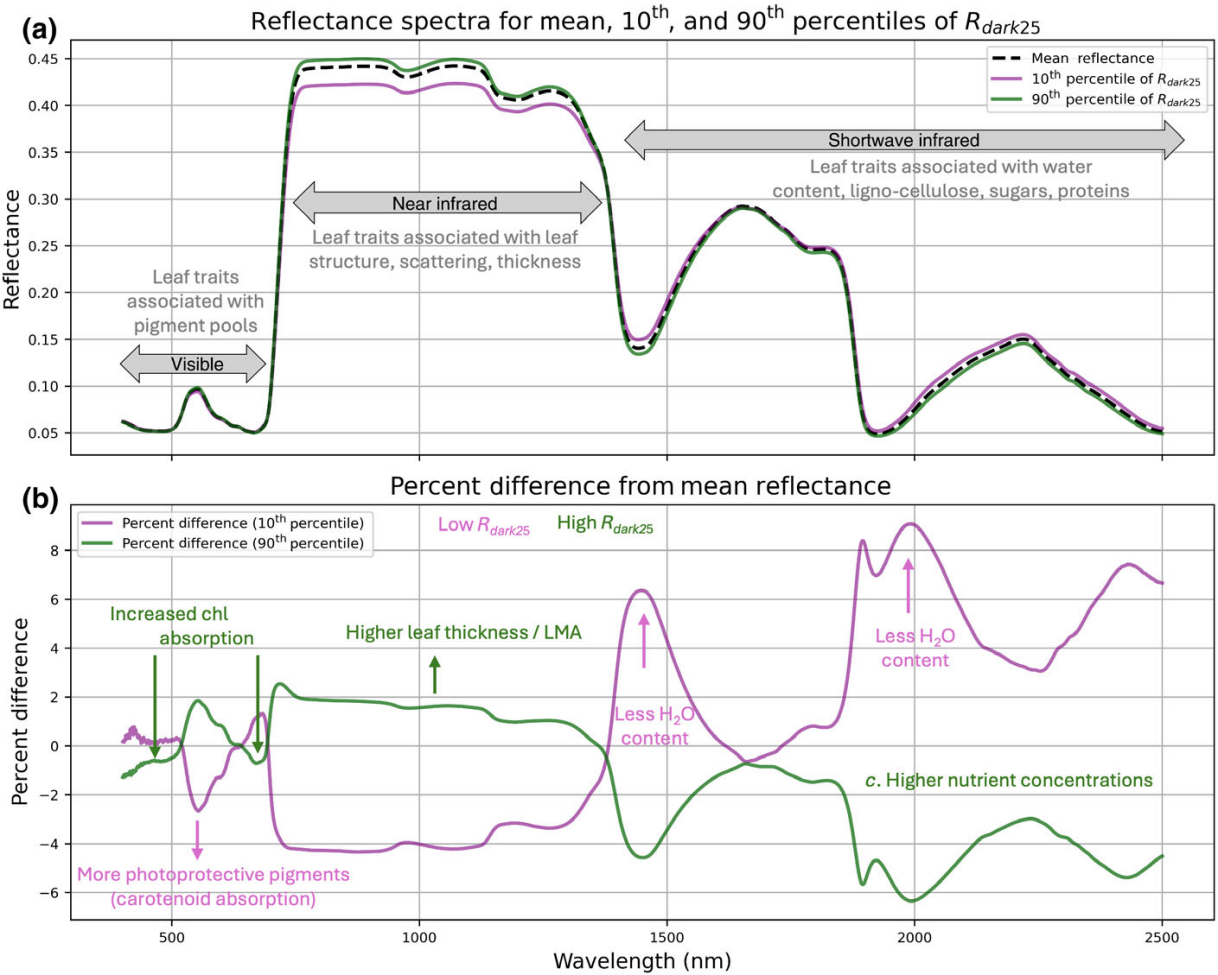
Subtle differences in hyperspectral reflectance curves from leaves representing a range of physiological conditions. (a) Reflectance data for the mean (50^th^ percentile, black dashed), high (90^th^ percentile, green), and low (10^th^ percentile, purple) *R*_dark_ samples from the Wu *et al*. (2025; pp. 481–497, in this issue of *New Phytologist*) dataset, highlighting key traits associated with regions of interest in the visible (400–700 nm), near‐infrared (*c*. 700–1400 nm), and shortwave infrared (*c*. 1400–2500 nm). (b) The percent difference between high‐ and low‐*R*_dark_ samples from the mean, annotated with the direction of observed differences for key traits. The high‐*R*_dark_ spectra show increased absorption in the chlorophyll (Chl) regions (*c*. 400–470 and *c*. 630–670 nm), while the low‐*R*_dark_ spectra show decreased reflectance centered at 531 nm, a prominent xanthophyll absorption feature. Additionally, there is greater reflectance in the near‐infrared region for high‐*R*_dark_ spectra, suggesting higher leaf thickness and leaf mass per area (LMA), and higher absorption (lower reflectance) in the water absorption features in the shortwave infrared.

The use of reflectance spectra to infer physiological activity has often relied on the principle that subtle changes in pigments are directly tied to photosynthetic processes (Gamon *et al*., 1992) due to the strong coupling between the light‐dependent and light‐independent reactions of photosynthesis (Magney *et al*., 2020). However, traditional optical remote sensing has primarily focused on the development of vegetation indices (VIs) – or combinations of typically two bands – which ascribe a unidimensional change with a specific plant function, similar to traditional leaf traits. Building on foundational work with VIs, researchers have expanded to using the complete hyperspectral reflectance spectrum (400–2500 nm) to improve the detection of plant physiological dynamics (Serbin *et al*., 2012; Barnes *et al*., 2017). The ability of hyperspectral reflectance to leverage multiple signals – ranging from pigments in the visible region (400–700 nm) to structural, nutritional and water‐related traits in NIR and SWIR – has enabled researchers to more accurately estimate photosynthetic capacity (Wu *et al*., 2019; Yan *et al*., 2021). This leap from traditional VIs to hyperspectral reflectance represents a paradigm shift in plant ecophysiology, unlocking the potential to track physiological dynamics across scales with greater precision and broader applicability.

Machine learning algorithms, such as Partial Least Squares Regression (PLSR), have been instrumental in identifying key spectral regions sensitive to photosynthetic parameters (Burnett *et al*., 2021). PLSR reduces the dimensionality of hyperspectral data by identifying latent components that summarize the relationships between spectral predictors and physiological traits. These components are linear combinations of the original spectral bands, designed to capture the maximum covariance between the predictors and the response variables (in this case, *R*_dark_). Unlike traditional regression methods, which use raw variables directly, PLSR transforms the data into a smaller, uncorrelated set of variables, minimizing noise and redundancy. Latent components in PLSR are derived from the entire spectrum, providing a holistic approach to capture subtle spectral signals linked to leaf traits, even those without direct absorption features such as Mg, Ca, and Mn. While these components lack direct physiological interpretations, they effectively summarize complex spectral patterns, making PLSR a powerful tool for predicting physiological traits.

Notably, PLSR remains an empirical approach, reliant on the relationships in training data. Models must be validated across diverse datasets and conditions to ensure their generalizability. Despite this limitation, the identification of key spectral regions through PLSR provides a foundation for understanding wavelengths of interest, offering a scalable alternative for ecosystem monitoring. This is done by plotting variable importance projections (VIPs) of spectral features (as in fig. 8, Wu *et al*., 2025). Here, many spectral regions identified by VIPs exhibit sensitivity to multiple traits due to their shared physiological roles. For instance, regions associated with Mg and N overlap with those involved in photosynthesis, structural integrity, and respiration, reflecting the interconnected nature of these processes. This shared variability allows spectroscopy to capture broad functional relationships among traits, enabling simultaneous monitoring of multiple leaf characteristics. However, it also introduces complexity in interpreting VIP scores, as the spectral bands may reflect indirect predictions based on covarying traits rather than direct mechanistic links (Wong, 2023).

One of the most compelling aspects of hyperspectral reflectance is its potential for generalization across scales and ecosystems (Serbin & Townsend, 2020). Metabolic parameters such as *R*_dark_ are plastic traits influenced by dynamic environmental drivers, meaning datasets must span diverse plant functional types, biomes, and seasonal gradients to improve model robustness. Wu *et al*. (2025) emphasize that addressing these gaps requires comprehensive datasets that account for vertical canopy profiles and full‐season dynamics (Niinemets *et al*., 2015; Lamour *et al*., 2023). These efforts will help bridge the trade‐off between site‐specific precision and cross‐site applicability, a critical balance for scaling plant processes optically. While most hyperspectral predictions of metabolic processes have been done at the leaf scale, its implications extend to larger spatial scales – from towers (Pierrat *et al*., 2024), to aircraft (Wang *et al*., 2020) to satellites (Cawse‐Nicholson *et al*., 2023). However, scaling introduces new challenges such as canopy heterogeneity, mixed pixels, solar/viewing angular effects, and background noise (Serbin & Townsend, 2020). Addressing these complexities requires hybrid modeling approaches that combine hyperspectral data with machine learning and radiative transfer models, as well as multi‐scale measurements (Pierrat *et al*., 2024).

Going forward, standardizing hyperspectral datasets across species and ecosystems is critical for using these methods at scale. For example, the development of open‐access databases such as the Global Spectra Trait Initiative (https://github.com/plantphys/gsti/tree/main), are essential for building a more robust framework. Ultimately, our growing need to rapidly detect change and track ecosystem function will benefit from tools that enable rapid, nondestructive, and scalable monitoring of plant metabolic traits. Hyperspectral reflectance bridges the gap between mechanistic understanding and large‐scale ecological monitoring, offering new insights into the drivers of carbon cycling and ecosystem dynamics. The recent paper by Wu *et al*. (2025) demonstrates the power of hyperspectral reflectance to capture a wider suite of leaf traits and move beyond the limitations of traditional unidimensional approaches, which fail to capture the complexity of plant physiological dynamics.

## Disclaimer

The New Phytologist Foundation remains neutral with regard to jurisdictional claims in maps and in any institutional affiliations.

## References

[nph20345-bib-0001] Atkin OK , Bloomfield KJ , Reich PB , Tjoelker MG , Asner GP , Bonal D , Bönisch G , Bradford MG , Cernusak LA , Cosio EG *et al*. 2015. Global variability in leaf respiration in relation to climate, plant functional types, and leaf traits. New Phytologist 206: 614–636. 25581061 10.1111/nph.13253

[nph20345-bib-0002] Barnes ML , Breshears DD , Law DJ , Van Leeuwen WJ , Monson RK , Fojtik AC , Barron‐Gafford GA , Moore DJ . 2017. Beyond greenness: detecting temporal changes in photosynthetic capacity with hyperspectral reflectance data. PLoS ONE 12: e0189539. 29281709 10.1371/journal.pone.0189539 PMC5744967

[nph20345-bib-0003] Burnett AC , Anderson J , Davidson KJ , Ely KS , Lamour J , Li Q , Morrison BD , Yang D , Rogers A , Serbin SP . 2021. A best‐practice guide to predicting plant traits from leaf‐level hyperspectral data using partial least squares regression. Journal of Experimental Botany 72: 6175–6189. 34131723 10.1093/jxb/erab295

[nph20345-bib-0004] Cawse‐Nicholson K , Raiho AM , Thompson DR , Hulley GC , Miller CE , Miner KR , Poulter B , Schimel D , Schneider FD , Townsend PA *et al*. 2023. Surface biology and geology imaging spectrometer: a case study to optimize the mission design using intrinsic dimensionality. Remote Sensing of Environment 290: 113534.

[nph20345-bib-0005] Gamon JA , Peñuelas J , Field CB . 1992. A narrow‐waveband spectral index that tracks diurnal changes in photosynthetic efficiency. Remote Sensing of Environment 41: 35–44.

[nph20345-bib-0006] Lamour J , Davidson KJ , Ely KS , Le Moguédec G , Anderson JA , Li QY , Calderón O , Koven CD , Wright SJ , Walker AP *et al*. 2023. The effect of the vertical gradients of photosynthetic parameters on the CO_2_ assimilation and transpiration of a Panamanian tropical forest. New Phytologist 238: 2345–2362. 36960539 10.1111/nph.18901

[nph20345-bib-0007] Magney TS , Barnes ML , Yang X . 2020. On the covariation of chlorophyll fluorescence and photosynthesis across scales. Geophysical Research Letters 47: e2020GL091098.

[nph20345-bib-0008] Niinemets Ü , Keenan TF , Hallik L . 2015. A worldwide analysis of within‐canopy variations in leaf structural, chemical, and physiological traits across plant functional types. New Phytologist 205: 973–993. 25318596 10.1111/nph.13096 PMC5818144

[nph20345-bib-0009] Pierrat ZA , Magney TS , Cheng R , Maguire AJ , Wong CYS , Nehemy MF , Rao M , Nelson SE , Williams AF , Grosvenor JAH *et al*. 2024. The biological basis for using optical signals to track evergreen needleleaf photosynthesis. Bioscience 74: 130–145.

[nph20345-bib-0010] Reich PB , Walters MB , Ellsworth DS , Vose JM , Volin JC , Gresham C , Bowman WD . 1998. Relationships of leaf dark respiration to leaf nitrogen, specific leaf area, and leaf lifespan: a test across biomes and functional groups. Oecologia 114: 471–482. 28307896 10.1007/s004420050471

[nph20345-bib-0011] Serbin SP , Dillaway DN , Kruger EL , Townsend PA . 2012. Leaf optical properties reflect variation in photosynthetic metabolism and its sensitivity to temperature. Journal of Experimental Botany 63: 489–502. 21984647 10.1093/jxb/err294 PMC3245480

[nph20345-bib-0012] Serbin SP , Townsend PA . 2020. Scaling functional traits from leaves to canopies. In: Cavender‐Bares J , Gamon JA , Townsend PA , eds. Remote sensing of plant biodiversity. Cham, Switzerland: Springer, 43–82.

[nph20345-bib-0013] Tcherkez G , Abadie C , Dourmap C , Lalande J , Limami AM . 2024. Leaf day respiration: More than just catabolic CO_2_ production in the light. Plant, Cell & Environment 47: 2629–2637. 10.1111/pce.14904 38528759

[nph20345-bib-0014] Ustin SL , Gitelson AA , Jacquemoud S , Schaepman M , Asner GP , Gamon JA , Zarco‐Tejada PJ . 2009. Retrieval of foliar information about plant pigment systems from high‐resolution spectroscopy. Remote Sensing of Environment 113: S67–S77.

[nph20345-bib-0015] Walker AP , Beckerman AP , Gu L , Kattge J , Cernusak LA , Domingues TF , Woodward FI *et al*. 2014. The relationship of leaf photosynthetic traits—*V* _cmax_ and *J* _max_—to leaf nitrogen, leaf phosphorus, and specific leaf area: a meta‐analysis and modeling study. Ecology and Evolution 4: 3218–3235. 25473475 10.1002/ece3.1173 PMC4222209

[nph20345-bib-0016] Wang Z , Chlus A , Geygan R , Ye Z , Zheng T , Singh A , Couture JJ , Cavender‐Bares J , Kruger EL , Townsend PA . 2020. Foliar functional traits from imaging spectroscopy across biomes in eastern North America. New Phytologist 228: 494–511. 32463927 10.1111/nph.16711

[nph20345-bib-0017] Wong CY . 2023. Plant optics: underlying mechanisms in remotely sensed signals for phenotyping applications. AoB Plants 15: plad039. 37560760 10.1093/aobpla/plad039 PMC10407989

[nph20345-bib-0018] Wu F , Liu S , Lamour J , Atkin OK , Yang N , Dong T , Xu W , Smith NG , Wang Z , Wang H *et al*. 2025. Linking leaf dark respiration to leaf traits and reflectance spectroscopy across diverse forest types. New Phytologist 246: 481–497. 10.1111/nph.20267 39558787

[nph20345-bib-0019] Wu J , Rogers A , Albert LP , Ely K , Prohaska N , Wolfe BT , Oliveira RC Jr , Saleska SR , Serbin SP . 2019. Leaf reflectance spectroscopy captures variation in carboxylation capacity across species, canopy environment, and leaf age in lowland moist tropical forests. New Phytologist 224: 663–674. 31245836 10.1111/nph.16029

[nph20345-bib-0020] Xu X , Trugman AT . 2021. Trait‐based modeling of terrestrial ecosystems: advances and challenges under global change. Current Climate Change Reports 7: 1–13.

[nph20345-bib-0021] Yan ZB , Guo ZF , Serbin SP , Song GQ , Zhao YY , Chen Y , Wu SB , Wang J , Wang X , Li J *et al*. 2021. Spectroscopy outperforms leaf trait relationships for predicting photosynthetic capacity across different forest types. New Phytologist 232: 134–147. 34165791 10.1111/nph.17579

